# ‘It feels like I'm back to being a teacher’: A longitudinal trajectory analysis of teachers' experiences during the first 8 months of COVID‐19 in England

**DOI:** 10.1111/bjep.12622

**Published:** 2023-06-20

**Authors:** Lisa E. Kim, Diana Fields, Kathryn Asbury

**Affiliations:** ^1^ Department of Education University of York York UK

**Keywords:** COVID‐19, longitudinal qualitative analysis, narrative identity, teacher professional identity, teachers

## Abstract

**Background:**

Understanding teachers' experiences throughout the school closures and reopenings that have characterized large periods of the COVID‐19 pandemic provides us with unique insights into what it means to be a teacher during a global public health crisis.

**Aim and Method:**

To investigate teachers' narratives of their experiences, we conducted 95 semi‐structured interviews with 24 teachers in England across four time points between April and November 2020. We used a longitudinal qualitative trajectory analysis of participants' stories of their high‐, low‐ and turning‐points.

**Results:**

We derived four themes that were evident at each time point and developed over time. The themes were: (1) *growing frustration at uncertainties caused by poor government leadership*, (2) *expanding concern for pupil learning and well‐being*, (3) *an increasingly labour‐intensive and exhausting job* and (4) *declining pleasure and pride in being a teacher*.

**Conclusions:**

The findings shed light on the impact of COVID‐19 on the professional identity of these teachers and we propose ways in which teachers can be supported now and in the future.

## BACKGROUND

The COVID‐19 pandemic affected the lives of many, including teachers. By late March 2020, schools had closed in 137 countries to help stem the spread of the virus (The World Bank, 2020). UNESCO (2020) identified 13 potential adverse consequences of these closures, three of which are directly related to teachers: confusion and stress; challenges around distance learning; and challenges around assessment. These risks were relevant to teachers in many countries, including England, the site of the current study.

By studying the experience of teachers throughout the public health crisis, we can examine whether and how the risks identified by UNESCO (2020) unfolded on the ground, and how they affected teachers. We can also gain valuable insights into their perceptions and beliefs about what it means to be a teacher, and how this has changed during the pandemic. These understandings are not only valuable in their own right but can also help countries understand how to preserve and nurture what makes being a teacher worthwhile and sustainable for those drawn to the profession (Rots et al., 2010). This is particularly relevant to countries with teacher recruitment and retention issues (Ovenden‐Hope & Passy, 2020).

### Teaching during COVID

**FIGURE 1:**
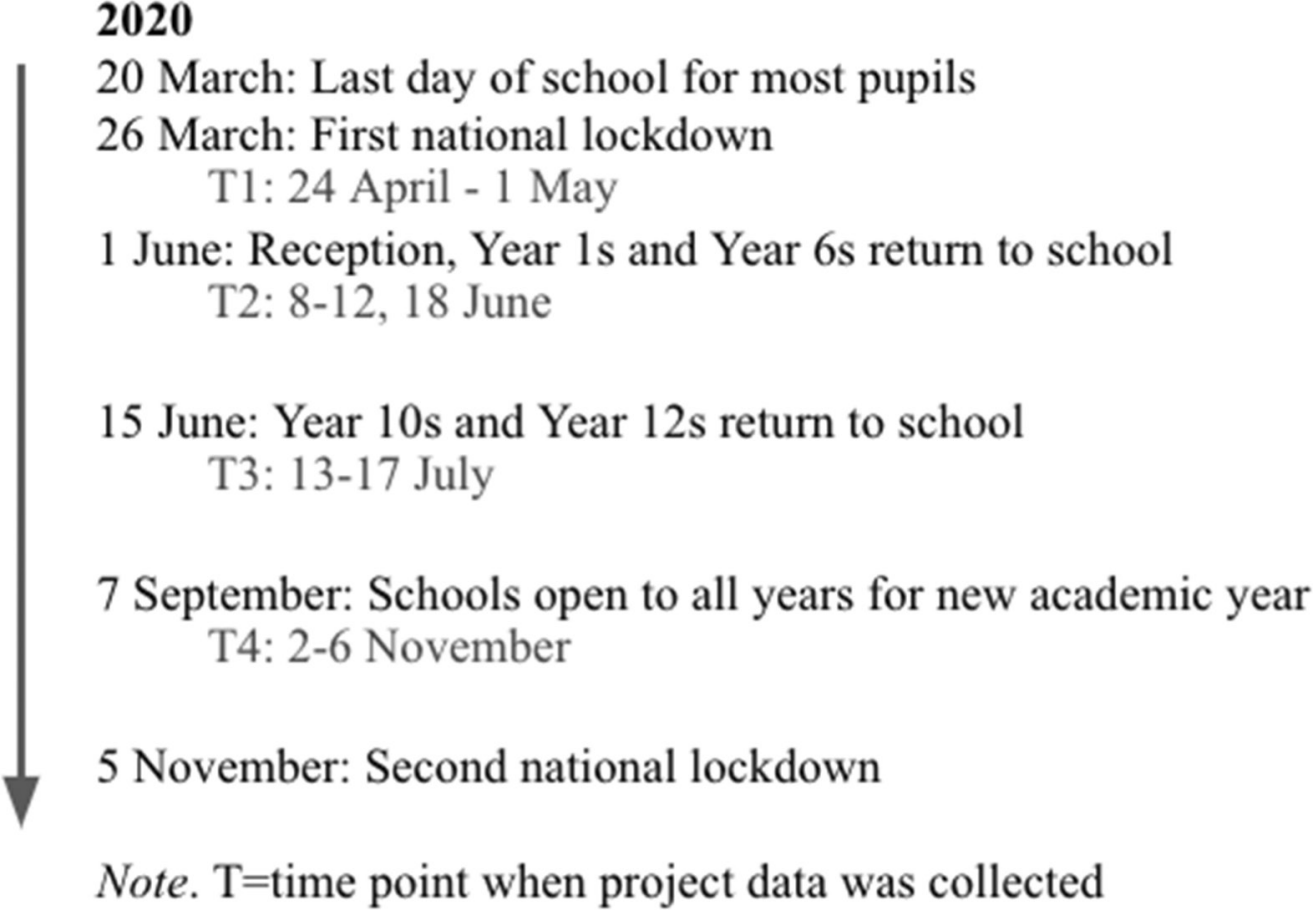
Timeline of 2020 school events and research data collection points.

Various key COVID‐19 events affected schools in England (see Figure 1): 20 March marked the last day of school for most pupils; 1 June marked the return of Reception, Year 1 and Year 6 pupils; 15 June marked the return of Year 10 and Year 12 pupils; and 7 September marked school opening to all year groups for the new academic year. Throughout these times of partial school closures, partial school reopenings and full reopenings, teachers continued to teach and look after the welfare of pupils.

During these times, teachers witnessed pupils struggle with difficulties that were often known about pre‐pandemic but these challenges, such as school attendance (McDonald et al., 2023) and transitioning from primary to secondary schools, presented differently during the unusual circumstances of the pandemic and required a new response. Teachers tried to assist where they could (Bagnall et al., 2022), all while they transitioned from a physical to a virtual world of teaching (Nazari & Seyri, 2021). The ‘exams fiasco’ in England also took a toll on teachers, as multiple U‐turns were made on how national public examinations should be handled (Kippin & Cairney, 2022). There was also evidence of general anger towards the government for a lack of clarity in the guidance they provided to schools and teachers (Young & White, 2022).

Moreover, teachers responded to the increased needs of their pupils and their families and went beyond the ‘traditional’ duties of a teacher to fulfil roles more typically associated with social workers and local community members or volunteers. That is teachers and schools individually monitored and responded to the pre‐existing and new well‐being and welfare needs of their pupils and their families, for example by delivering food parcels (Kim, Dundas, et al., 2021; Moss et al., 2020). Although cases of the need to fulfil such duties have been noted in the past (e.g., Gilligan, 1998), the prevalence and the need for them grew during the pandemic.

These circumstances and experiences may have affected multiple aspects of teachers' lives. Concerns about teacher well‐being are inevitable as they experienced an imbalance between job demands and job resources which, according to the Job Demands–Resources Model (Bakker & Demerouti, 2007), is likely to have negative effects on their levels of work engagement and potential for burnout. From a wider meaning‐making perspective, their experiences of intensified and extended work in these difficult times may have led to questioning and re‐negotiating their professional identity; that is, what it means to be a teacher (Mockler, 2011).

Teacher professional identity is a lens through which teachers can view, interpret and construct meaning regarding what they do and their place in society (Sachs, 2005). It is a malleable construct that is constantly being shaped and renegotiated over time (Kerby, 1991). Various theoretical models of teacher professional identity have identified factors that contribute to its formation and development. For example, Beijaard et al. (2004) propose that an interaction between factors on two axes (individual/collective; public/private) contributes to the formation of teachers' professional identity, while Suarez and McGrath (2022) identify factors at the individual, professional and contextual levels (see Suarez & McGrath, 2022 for a review). What is clear across these models is that factors which range from the individual to the system level, and the interactions between them, can all contribute to the professional identity among teachers. Moreover, the roles and responsibilities that teachers need to fill have diversified over recent decades, for example in response to a shift to more student‐centred pedagogies (Schleicher, 2018), and increasing digitisation (Engeness, 2021). These developments have increased pressure on teachers and increased the potential for confusion regarding their professional identity.

Many aspects of these contributing factors indeed changed as a result of the circumstances instigated by COVID‐19, which in turn would have required teachers to continuously negotiate and renegotiate their professional identities. Moreover, as Suarez and McGrath (2022), propose in their teacher professional identity development and outcome model, teachers' professional identity can reciprocally affect their behaviours and attitudes (e.g. teaching quality and commitment and intention to remain in the profession), and thereby pupil outcomes (e.g. learning and attitudes). For example, one of the rare studies of teachers' professional identities during COVID‐19 found that the professional identities of five primary school teachers in Ireland were constructed and reconstructed, marked with positive growth and commitment and re‐negotiations of how to work with pupils and parents (Mellon, 2022). However, the potential changes in teachers' professional identities and their associated consequences for their behaviours and attitudes over time remain unknown. The model by Suarez and McGrath (2022) model would predict that challenges in the individual, professional and contextual realms would have a negative effect on professional identity and, thereby, on outcomes such as reduced teaching quality and commitment to the profession.

### Narrative identity theory

Storytelling is a common way to capture teachers' professional identity (see Beijaard et al., 2004 for a review). Specifically, a narrative identity framework is helpful to understand teachers' professional experiences and the ways in which they draw meaning from those experiences. Narrative Identity Theory (McAdams, 2001; McAdams & McLean, 2013) suggests that individuals develop and internalize an evolving self‐story based on autobiographical memory. These subjective life story narratives develop with experience and they help individuals to make sense of their experiences and to organize them into a coherent narrative self, which thus provides them with an on‐going unity and sense of purpose (McAdams, 2001). Teachers' self‐stories during COVID‐19 are likely to be integrated into their broader life stories, making them relevant to their long‐term personal and professional narratives, as well as to our understanding of the impact of the pandemic. Others have also argued that teacher professional identity is generated through narratives (Hamilton, 2010), and have emphasized the importance of taking context into account in understanding such narratives (Edwards & Miller, 2007; Menter, 2008). In this case, we examine the context of a global pandemic and the way it unfolded for teachers in England during 2020.

By asking teachers to share multiple self‐stories (high‐, low‐ and turning‐point scenes) from their professional experience during COVID‐19, we can observe their subjective priorities and comparisons of current and previous experiences during the dynamic process of developing their narrative self and professional identity. COVID‐19 offers us a unique opportunity to study teachers' self‐stories during a period of major disruption and to explore the implications for supporting teachers now and in the future.

### The current study

We present self‐stories from teachers in England captured through semi‐structured interviews at four time points between April and November 2020, which mapped on to key COVID‐19 events for schools in England. By giving participants the freedom to select their scenes and describe them without constraints, we can observe what was subjectively most important to them and identify potential support mechanisms.

We used the interview data to ask: *What was it like to be a teacher in England during the first 8 months of the COVID‐19 pandemic* (i.e.*, different stages of partial school closure to full school reopenings*)*, and how did the experiences change over time?*

## METHOD

### Participants

We conducted 95 interviews with 24 teachers across four time points (Ts) in 2020 (see Figure 1): 24 April to 1 May (T1); 8–12, 18 June (T2); 13–17 July (T3); and 2–6 November (T4). All 24 participants took part in T1–T3 and one withdrew from the study at T4. The participants were state school teachers in England (11 primary and 13 secondary; 6 male and 18 female) with a wide range of teaching experience (1–32 years; *M* = 12.55, *SD* = 8.94). In England, the ages of pupils in primary schools range from 4 to 11 (Reception to Year 6), and in secondary schools from 11 to either 16 or 18 (Year 7 to Year 11 or Year 13 if the school has a sixth form). Participants worked in the North, South, East and West of England, and in schools with the full range of Index of Multiple Deprivation deciles (a relative measure of deprivation in England; Ministry of Housing, Communities, & Local Government, 2019).

Similar to Gu and Day (2007) categorisation, we identified participants as Early Career Teachers (ECT, ≤5 years of experience); Mid‐Career Teachers (MCT, 6–18 years of experience); and Late Career Teachers (LCT, ≥19 years of experience). We classified teachers who were members of senior leadership teams (SLT), such as headteachers, deputy/assistant headteachers and Special Educational Needs and Disabilities Coordinators (SENDCos) as SLT. When presenting quotations we provide participant number, school type (primary or secondary), career stage and time point as context. For example, ‘P1, primary SLT, T1’ refers to Participant 1, an SLT member in a primary school, speaking at time point 1.

Participants were recruited via social media and email, and the final sample was selected to ensure roughly equal representation of primary and secondary school teachers; and early‐, mid‐ and late‐career teachers and SLT members. All participants were compensated with a gift voucher at each time point, receive copies of the project outputs, and have been regularly invited to provide feedback to the ongoing study. Ethical approval for the study was granted by the researchers' university department.

### Measures

Data were gathered using a semi‐structured interview schedule adapted from Section B of the Life Story Interview (LSI; McAdams, 2008). We adapted the LSI to ask participants to identify and describe three key scenes from their experience of being a teacher during a global pandemic: a low‐, high‐and turning‐point. We asked participants to share these three stories at each time point, providing as much detail as possible about when and where the scene took place, what happened, who else was there and what they were thinking and feeling, and to reflect on what their choice of scene might say about them as a teacher (i.e., to draw meaning from their own narratives). The same wording was used at all four time points for consistency.

### Procedure

Interviews took place on Zoom to meet the social distancing requirements that were in place at the time. Participants were interviewed by the same researcher at each time point. At the start of each interview, participants were reminded that they could withdraw at any point, and how their anonymised data would be used. The interviews were recorded, generating automatic transcripts, which were edited and anonymised prior to coding and analysis. Most of the interviews for each time point took place within a single week, ensuring the participants were at the same stage of the school year and pandemic.

### Coding and analysis

Our interest was in identifying stability and change in teachers' self‐stories over time during COVID‐19, and in the meanings they drew from them in relation to their professional identity. Taking a recurrent cross‐sectional approach (Grossoehme & Lipstein, 2016) we coded and analysed the data at each time point using reflexive thematic analysis, a theoretically flexible approach that suited both our research question and sample size (Braun & Clarke, 2021). We followed up this series of four synchronic thematic analyses with a diachronic analysis that focused on how the experience of being a teacher changed over time for these participants (Grossoehme & Lipstein, 2016; Neale, 2021; Saldaña, 2003). The diachronic analysis is the focus of the current paper.

Coding of the cross‐sectional data was both manifest and latent but primarily manifest as we were interested in the overt content of teachers' self‐stories. T1 transcripts were coded inductively as COVID‐19 represented a completely new situation (Kim & Asbury, 2020). At subsequent time points the transcripts were coded both inductively and deductively. That is, the codes and themes were generated separately for each time point (T2–T4) and deductive comparisons and adjustments were made with previous time points to ensure consistency in code clustering and theme labelling. Some abductive logic was applied as we iteratively considered findings from each time point in relation to the theoretical literature and in relation to each other (Neale, 2021).

At each time point, data extracts were gathered to support each code and all codes were discussed within the team, with refinements being made on the basis of these discussions. Themes were generated by clustering codes in a variety of ways until a solution was reached that all authors agreed accurately represented the data. This happened separately for each time point and we were mindful to ensure that equivalent ideas or experiences at different time points were coded using the same labels, to support the spotting of patterns over time. We prepared group‐based pen portraits of the data at each time point that formed a starting point for the diachronic analysis.

Once a full reflexive thematic analysis had been conducted for each time point and a pen portrait prepared, we turned to our diachronic longitudinal trajectory analysis. The process was primarily based on Grossoehme and Lipstein (2016) approach. We revisited the stories data at each time point and the pen portraits we had developed on the basis of it. By immersing ourselves in the data we were able to identify connections between each time point and to take a temporal approach. In considering longitudinal themes we were guided by questions from Saldaña (2003) including framing questions (e.g. What is different from one pond or pool of data through the next?); descriptive questions (e.g. What increases or emerges through time?) and analytic/interpretive questions (e.g. What is the through‐line of the study?). This approach allowed us to reflect on the temporal nature of the data and to address our research question which is focused on continuity and discontinuity in teachers' experiences over the course of the first 8 months of the COVID‐19 pandemic. We used abductive logic in moving back and forth through the four datasets and between the data and the theoretical literature (Neale, 2021). This allowed us to develop a set of longitudinal themes that were refined through a process of team discussion and iteratively revisiting relevant theoretical literature and the data.

### Positionality and reflexivity

Our analysis was conducted from the perspective of critical realism, focusing on teachers' observations of the unfolding events that characterized the first 8 months of the COVID‐19 pandemic in England. Other factors are also likely to have influenced our analysis. For example, one of the authors had a long career in teaching prior to becoming a researcher and is likely to have brought this experience to the analysis. One is a parent whose family was directly affected by school closures and home‐based education during COVID‐19. It is likely that these experiences coloured our interpretation of the data to some extent. However, we worked as a team to ensure agreement around the interpretations we made. Any areas of disagreement triggered team discussions until a way forward could be agreed that made sense to everyone.

Although all participants were given access to our publications as preprints, including this one, and were invited to share feedback on them, we did not engage in more proactive member checking. This was largely because we were already asking a great deal of our participants with very regular interviews at an unprecedentedly busy time. We believe that participants felt well represented by our presentation of findings as we received no responses suggesting otherwise, but this cannot be known with certainty.

## RESULTS

The four longitudinal themes identified across the four time points were: (1) *growing frustration at uncertainties caused by poor government leadership*, (2) *expanding concern for pupil learning and well‐being*, (3) *an increasingly labour‐intensive and exhausting job* and (4) *declining pleasure and pride in being a teacher*. These themes build on our cross‐sectional, synchronic descriptions and focus on how experiences of being a teacher and professional identity developed over this 8‐month period, drawing out the longitudinal stories that were most strongly identified in the data.

### Theme 1: Growing frustration at uncertainties caused by poor government leadership

Uncertainty was dominant in teachers' low‐point stories, particularly at T1 when the pandemic first began. It was also a feature of some turning‐point stories but never of high points. There was a clear link between participants experiencing uncertainty about how to do their jobs, given the varied and changing constraints imposed by COVID‐19, and their experiences of stress and anxiety at all time points. However, the nature of that stress and anxiety changed over time, moving from acute to chronic, and was increasingly linked with frustration with government leadership.

There is no doubt that these teachers were experiencing acute uncertainty about how to do almost every aspect of their job at T1, and that this was linked with very high feelings of stress and anxiety. As P24 (secondary LCT, T1) put it: ‘I guess it felt a bit like, you know, you're shown the diagram of how the parachute works and then you're pushed out of the plane’. However, there was no sign at this stage that participants felt they were experiencing higher levels of uncertainty than others, or that anything could be done about it.

This changed at all subsequent time points, but particularly T2 and T4, as participants increasingly blamed the government for the level of uncertainty that schools and teachers were experiencing, and the stress that it caused them. They described being subjected to a relentless procession of barriers to being able to do their jobs, and many felt that this could have been avoided had the government employed better communication strategies, greater clarity and some compassion and respect towards the profession. P11 (primary ECT, T2) described a perceived lack of government trust as a reason for poor communication and disrespect:

the government never said, we're thinking of opening. What do you think the barriers are going to be or what do you think the challenges are going to be? And there was never that dialogue or that trust there really and that kind of… I just don't think they trusted the teachers with opening, they said we're opening on this day.

With regard to government guidance, P15 (primary LCT, T2) said: ‘We were bombarded with risk assessments and this procedure and that procedure, and this policy has changed and that policy has changed, and none of us quite knew what we were going into’. An ongoing concern was with the government announcing what would happen in schools to the general public without any advance warning. P1 (primary SLT, T2) said:

I think what is unfair is that we find out what's happening in schools when the general public do, but then there is a very short timescale … I just think it's unreasonable. I think the government haven't thought about the wellbeing of teachers throughout this time. At all.

The increase in frustration with the government eased a little at T3 with some participants appreciating the timely announcement of the plan for full reopenings in September, even while being intimidated by the work it would entail. Some participants welcomed it as an end to uncertainty. P19 (secondary MCT, T3) said: ‘It was nice to have some concrete plans of what they envisage it being like’. However, others had already lost trust that this would be the case. P9 (secondary SLT, T3), for example, spoke of SLT meetings spent ‘planning for September which, personally, I think is a total waste of time because we're going through the guidance clause by clause … and you know eight weeks down the line it's going to be totally different’.

Frustration at uncertainty was huge at T4. This was partly caused by ambivalent guidance on issues such as whether pupils should wear masks. P6 (secondary SLT, T4) described their anger at a lack of leadership on this issue, which they perceived as a deliberate tactic to ensure the blame for any wrong decision would be placed at the doors of school leaders rather than the government: ‘Tell us, tell us what you want us to do. And then I'm really happy to crack on and do it. I can't stand this, you know, middle ground, that actually you're being judged on’. However, the main cause of frustration was the announcement, just before our T4 interviews, that there would be a second national lockdown but that schools would remain open. P4 (primary SLT, T4) said:

In the last 48 hours the country has been put into a second lockdown. And once again we've had no preparation, no warning, no guidance at all. We've just been told that schools will be open and at no point will we be closed.

P3 (primary SLT, T4) added that it was: ‘so hard … to have to try and keep a sense of normality when it's anything but and it feels almost like the rest of the country has been called to stop what they're doing and keep themselves safe’. This uncertainty, around what was expected of them but also the potential risk to their own health and that of their pupils and their own family members made planning extremely stressful.

In each of these instances, participants believed that the uncertainty they experienced was unnecessary and almost always due to poor leadership from the government. It led to growing frustration and resentment, and to participants feeling ground down by the barriers put in the way of them being able to do their jobs effectively. P23 (secondary LCT, T2) bemoaned how ‘every day, the information comes out, which is contradictory to the week before, or the day before, even, and it's very difficult to plan’.

Our data suggest that teachers wanted, and expected, to be well managed and to have infrastructure and processes in place that would allow them to function effectively as teachers. As one school leader told us: ‘either work with us or against us and at the moment you're just working against us’ (P4, primary SLT, T4). While participants could understand this not being possible at T1 – when everybody was just trying to find their feet – they felt let down by government leaders not ensuring sufficient stability for schools and teachers to be able to plan effectively at subsequent time points, and felt this was driven by a lack of respect for the profession. P1 (primary SLT, T2) said: ‘the government haven't thought about the wellbeing of teachers throughout this time’. P16 (secondary ECT, T4) described feeling very happy to be back in school ‘but still so utterly let down’.

One particular example of this frustration related to the way in which high‐stakes assessments at ages 16 and 18 were managed, and the implications of perceived mismanagement from government for both teachers and pupils. From the outset, teachers were worried about disruption to their exam classes, all due to take assessments at the end of 2 or 3‐year programmes of learning in May and June 2020. P9 (secondary LCT, T1) said: ‘I don't think anyone will ever appreciate the amount of effort that's gone into getting these grades right’. To some extent, worries were assuaged when the decision was quickly made that pupils would be graded on the basis of teacher assessment and would not sit formal exams. Given this, the government's decision to moderate teacher‐assessed grades with an algorithm designed to combat potential grade inflation triggered controversy and high levels of frustration for teachers, particularly when results were made available in the summer. Some teachers described sadness that their pupils were being let down by grades they considered unfair and unrepresentative. A swift U‐turn, in which pupils were awarded their unmoderated grades, was welcomed but also added to teachers' frustration. At T4 P7 (secondary SLT) reflected: ‘everybody was a bit up in the air and it felt like we'd let the students down very much because we should know what's happening’.

Government leadership of public assessments during this period represents a good example of teachers' experience of being on a rollercoaster where at points there seems to be a good solution in sight and then something changes that leads to another dip in morale and increase in stress and worry for pupils. This frustration was exacerbated by a feeling that the failed algorithm represented government distrust that teachers would do their jobs fairly and well, a feeling that was felt much more broadly than in the context of assessment. P4 (primary SLT, T3) explained: ‘You come back to this … overriding impression that teachers and schools and headteachers are not trusted as professionals … they wouldn't do that to other professions; they wouldn't treat the medical profession like that, there'd be uproar’.

In summary, these teachers accepted that COVID was a new and unfolding situation, and accepted that there was some uncertainty baked into that. At T1 the uncertainty was huge but it was the same for the rest of the country and there was a recognition that this was a time for pulling together, not for politics. However, by T2 teachers were becoming increasingly frustrated by, and angry at, poorly thought out and poorly communicated guidance as well as perceived professional disrespect. This stayed the same or even eased a little at T3 when many teachers were back in school at least some of the time. They actively appreciated being told the plan for September well in advance, although trust was too broken for some to believe it would not change. However, frustration was dominant again at T4, partly around issues with the quality and feasibility of government guidance but very significantly because of the decision, without consultation, to keep schools fully open while the rest of the country went into a second national lockdown.

### Theme 2: Expanding concern for pupil learning and well‐being

Participants' worries increased over the course of the first 8 months of COVID‐19 and related to a widening group of pupils as the situation persisted, particularly at T2 and T3. Worry for pupils was a dominant theme in low‐point stories at all time points. It was clear that participants felt a significant level of responsibility for their pupils' learning and well‐being, and that they genuinely cared about them. Their stories suggest that caring, and a sense of responsibility, are core elements of teacher identity, and of the day‐to‐day job. This is something that was stable throughout the period but we saw changes in who, as well as what, participants worried about, as the situation persisted.

At T1 many of the teachers were distressed, and sometimes even scared, by the situation their most vulnerable pupils found themselves in. Most worries focused on safeguarding those children known to be at risk as well as the most economically disadvantaged of them. As P2 (primary SLT, T1) put it: ‘the worst bit is just not being able to see some of the pupils that we know are really, really vulnerable’. P12 (primary ECT, T1) described how: ‘There is a girl in my class who there are safeguarding issues around … When there's an opportunity for her to come in the family go into self‐isolation’. Worries like this were exacerbated by the uncertainty described in Theme 1.

But when we're away from that child, and we can't have direct contact with them, then we don't know what effect that incident is having or had on that child and we know that that child is still in a potentially volatile situation. And I think for us that are safeguarding, not being able to safeguard in the way we always have, I think that is the hardest thing for us. (P2, primary SLT, T1)

As time went on participants continued to worry about these very vulnerable children and generally felt happier when they were able to be back in school. Even at this early stage though, they noticed that the number of children they needed to worry about was growing. For example, P11 (primary ECT, T1) described how:

I got an email to say that a child was coming to school that isn't a key worker child because they're now a vulnerable child because of something that happened at home which meant they were no longer safe at home … it's changing the dynamics of stable homes. I just think that's really shocking.

As the COVID‐19 situation settled, and became embedded, more participants became worried about the ‘newly vulnerable’ (P7, secondary, T3). These included pupils with new safeguarding concerns but also a broader group. For example, P11 (primary ECT, T2) described reading with a 5‐year‐old girl who had been viewed as a high achiever before COVID‐19, saying: ‘it was a book that she would have read really easily early on in the year and she really struggled with it, and it showed me that even those children who aren't vulnerable … [are] falling behind’. Of course they also remained concerned about the pupils they had always known to be vulnerable. For example, P10 (primary ECT, T2) talked about one child from an economically disadvantaged family who has special educational needs and speaks English as an additional language, saying: ‘there was no contact at all … and so that was quite a scary thing really’. They added: ‘I always think that the Mum was, kind of, not blocking our calls but as soon as she saw our number or an unknown number … she was just hanging up’.

A major new concern at T2, particularly for secondary school teachers, was a lack of engagement with the education they were providing. P19 (secondary MCT, T2) said: ‘the take up is quite low. And it's just depressing … I don't know how we engage and support them’. We saw a small number of teachers begin to blame themselves for low engagement at this point, worrying about the implications for their pupils. For instance, P20 (secondary MCT, T2) described how at the start of the pandemic they had focused on making work available, rather than on the pedagogical basis for the work, largely because they did not know how long to expect the situation to last. In reflecting on very low rates of engagement they said: ‘I wonder if that's when it started and, therefore, that feels … like a really stupid thing for us to have done … that had a catastrophic effect’.

Concern about low engagement and the learning loss associated with it, was even more dominant at T3, with P3 (primary SLT, T3) saying ‘pretty much most weeks it's 10% or less’. P3 (primary SLT, T3) said: ‘It's bad because … the children, by the time they come back … in September, potentially they've had almost six months of time away from learning and away from routines; away from structures; away from being motivated and switched on’. P11 (primary ECT, T3) felt that those children who had been able to access school, such as key workers' children, were at an advantage: ‘Those children have really flourished … And then it makes you wonder about the ones who are not in school, and what their wellbeing's like, and how well they're doing academically’. Participants described worries about the learning and well‐being of all pupils, but particularly those with whom they had little or no contact.

By T4 all children were back in school and would continue to attend, even through a second national lockdown. This alleviated most concerns about most pupils' well‐being, at least as it related to the pandemic situation, but worries about lost learning continued. P14 (primary MCT, T4) said: ‘It's had a massive impact on them … I'm in Year 5 but I feel like maybe I've gone back a few years’. We also noted worries about the limited school experience they were able to provide. P19 (secondary MCT, T4) said they would be angry if they were the parent of a child starting secondary school:

I just really feel for the Year 7 students … they're not set by ability, haven't got their SATs results. We should be able to push them really hard, the high ones, and really support and nurture the ones that struggle and we just can't. And it's just horrible.

In summary, we observed how acute worries around safeguarding and the most vulnerable children dominated T1 stories. This remained a concern whenever COVID‐19 restrictions made it hard to provide support but from T2 we saw more of an emphasis on engagement and learning. In some cases, this triggered self‐doubt about the learning opportunities that schools and teachers had provided. For many participants, worries reduced in September when all children were back in school but some remained concerned about lost learning and their pupils' educational experiences.

### Theme 3: An increasingly labour‐intensive and exhausting job

Teaching was a demanding job long before COVID‐19 and, over the course of the first 8 months of the pandemic, participants described how the demands ramped up leaving them exhausted, dispirited and, in some cases, completely hopeless. By T4, when all schools had been fully re‐opened for one half term P16 (secondary ECT, T4) said: ‘I've never been so stressed in my life … the workload … is increasing exponentially’ and P4 (primary, SLT, T4) said: ‘Exhausted. I'm emotionally exhausted, I'm physically exhausted … And I think to a certain extent now I'm psychologically exhausted as well’. There were clear signs in the data that by T3, and certainly T4, these teachers were at a very low ebb, with some edging close to burnout. As P1 (primary SLT, T3) put it: ‘There are times when you just go, I can't do this. Does that make sense? It's just another thing on top of another thing or something else. You just think, what next?’

There were two main reasons why teachers and school leaders had become so tired by this point. The first was that there was simply more work to do with, for example, teachers being required to teach in‐person classes at the same time as teaching pupils who were at home due to COVID‐19 infections. The second was that every change during this period, from almost full school closures at T1 through to full reopenings at T4 triggered a raft of new demands, policies and skills to learn.

The simple fact of there being more work to do was pointed out at every time point, although a minority of classroom teachers felt they had more time at T1 and, in spite of everything, enjoyed a brief period of improved work‐life balance compared to their pre‐pandemic routine. As one early career teacher put it: ‘I'm actually quite enjoying just having that time to look at the children's work online, having that time to catch up on paperwork and just relax a little bit’ (P11, primary ECT, T1). This was unusual though and had almost completely disappeared by T2 when schools were preparing for partial reopenings. P17 (secondary ECT, T2) reflected:

I think in terms of turning point for me it was definitely not long after we last spoke because I remember quite vividly saying I feel like my workload is so much shorter … and, typical karma would get me for that, because in a week's time I had so much going on.

At T2 the plan to open school to more pupils generated increased work for all teachers. but particularly for senior leaders. In describing the announcement, on 24th May, that some groups would return to school buildings from 1st June, P1 (primary SLT, T2) said: ‘the turning point … is Boris' announcement on that Sunday because, since that moment in time the … rush to get everything ready, everything prepared, the amount of paperwork that came after, that work was horrendous … a logistical nightmare’. They added: ‘I don't think I've ever worked so hard in my life if I'm honest, in … the week before we opened’.

Some T2 stories were characterized by pride at how hard school communities had worked to prepare school buildings for children and young people to return in June 2020. P7 (secondary SLT, T2) said: ‘You see the members of the community there, the cleaners, the catering staff who are … keeping free school meals going. The … site team. Oh my god, they've had no time off’. It is clear that an increase in workload was felt by all at T2, particularly those who were members of SLTs and perhaps even more particularly those who were members of primary SLTs. P4 (primary SLT, T2) said: ‘I've hoovered the floor, cleaned the toilet. I've, you name it I've done it. You know, I can't sit in my posh office’. P1 (primary SLT, T2) corroborated this: ‘Some days I've been more of a janitor than I have a senior leader’.

At T3, the demands ramped up further still. The full reopening of schools is something that participants viewed very positively, something they had longed for during the early months of the pandemic. Once again though, their positivity was tempered by relentlessly increasing job demands. And, once again, SLT participants appear to have been particularly affected. ‘I've got to rewrite every risk assessment … evaluate everything … update everything on the website … you know, take the staff through yet another upheaval’ (P4, primary SLT, T3). Primary SLTs were affected by having smaller teams with which to share the workload, particularly in small schools. P2 (primary SLT, T3) commented: ‘I think it's knowing that I am not working at my best ability because I am exhausted … I'm not sure I can carry on doing this’.

While the sudden increases in demand appear to have been strongest for those in senior leadership positions at T2 and T3, by T4 the change of pace was such that everybody was affected:

So we're doing the usual madness of September … and then added on top of that … we've had to set things up because of coronavirus, so extra things in class, things that are taking longer because we've had to do washing hands and all the rest of it, and having to also work online in case [a] child [is] self‐isolating and then having to learn new things like how to do parents evening via Zoom. (P15, primary LCT, T4)

Ever‐increasing demands make it unsurprising that by T4 all participants were so tired. P20 (secondary MCT, T4) said: ‘That pace was really hard and I crawled towards half term. We're exhausted’.

Overall we observed pride in stepping up to the plate and meeting the demands of the situation at T1 and 2, and even at T3 although teachers were increasingly ground down by this point. By T4 the majority of participants were extremely tired, overwhelmed by ever‐increasing demands and only one‐half term into an academic year during which COVID‐19 would continue to exert a very significant influence on the experience of being a teacher. The effect of increasing job demands was exacerbated by ongoing uncertainty about a situation which had proved highly unpredictable during the first 8 months of the pandemic. P14 (primary MCT, T4) concluded: ‘I don't think that this is maintainable, the actual workload that we're doing at school. We're at school stupid amounts of time … I would consider a job doing anything else’. During a teacher recruitment and retention crisis, conclusions such as this are troubling.

### Theme 4: Declining pleasure and pride in being a teacher

Participants lost much of what it meant to be a teacher at T1 when most found themselves abruptly unable to be in classrooms with their pupils and having to work in a completely new way. As P24 (secondary LCT, T1) put it: ‘your training didn't prepare you for teaching online’. For many, this triggered a decline in enjoyment of their job which was not reversed by T4 in spite of everyone being back in school and a return to something closer to normality by then.

The primary loss described at T1 was of the relationships that participants described as being core to their identity as teachers (i.e. with pupils). As P2 (primary SLT, T1) put it: ‘we all thrive on that … interaction and that connection with … kids and that wanting to help and support and nurture them and being not able to do that is … really hard’. They also lost easy access to the relationships they saw as the best sources of professional support for themselves (colleagues). Although they maintained their relationships with pupils and colleagues online they also saw those online relationships as pale imitations of the real thing: ‘it's hard to replicate natural conversation online’ (P24, secondary LCT, T1). The participants who appeared to take the biggest knock to their professional identity at this stage were those who could not be in school in the days before lockdown, either due to a suspected COVID infection or being clinically vulnerable. P23 (secondary LCT, T1) described feeling: ‘I wasn't needed. Many teachers were surplus to requirements because of the low numbers of key worker children who were in schools … because of my pre‐existing health conditions they said, no, just no, we can manage without you’. These teachers found it particularly difficult that they were not able to say goodbye to their pupils:

I called my manager and she said that I wasn't allowed to come into school obviously because my son had a symptom and it just felt like I'd … been ripped apart from my career almost because I couldn't see the kids … I couldn't say goodbye to them. (P5, secondary ECT, T1)

However, we also noted evidence of pride in doing a good job in exceptional circumstances at T1, particularly for SLT participants. P3 (primary SLT, T1) described how ‘we were calm in the crisis. It wasn't like headless chickens. We were swans swimming you know, paddling, paddling like crazy underneath but serene and calm on the surface’.

At T2, as at all four time points, participants expressed the most pleasure at any opportunity to be in school, albeit with small groups of children. P11 (primary ECT, T2) described how: ‘my biggest high point was definitely … going back to work’. However, many also described feeling dispirited as a result of low contact and limited engagement. P18 (secondary ECT, T2) said: ‘you feel that you're not really contributing to your school environment. You're doing all the online stuff but … it almost felt quite pointless because you got literally no responses from students, or very, very few’. There was also a feeling that initial good will towards teachers was waning, and this was supported by negative communications from some parents. P6 (secondary SLT, T2) described receiving an email from a parent while they were working every day of half term: ‘basically it's saying that nobody in the school has done anything and we're all rubbish’. P4 (primary SLT, T4) also commented on the turning tide of public opinion: ‘there's kind of a lack of understanding from the general population … about what's happening in schools and how we are expected to work and the stresses and strains that we are under … like a really serious lack of comprehension’. This perceived negativity had a clear negative impact on participants' professional pleasure and pride.

T3 was at the end of the academic year, just before the summer holidays, and participants noted it being an unusually flat end to a school year. P11 (primary ECT, T3) commented that: ‘normally it's such a lovely experience celebrating the year, and I mean we still did that, but it was with only a handful of children’ and P12 (primary ECT, T3) added: ‘probably the low point as a teacher during this time of year is not being able to say goodbye to your current class’. Some participants reflected on how the pandemic had changed them, with P2, primary SLT, T3 saying: ‘I've lost a lot of the empathy I normally have … It's not the person that I want to be. It's not the teacher I want to be. It's not the leader I want to be’. They also commented on how little they had enjoyed remote teaching, an ongoing reality for most of them: ‘your job isn't just sat at the computer sending emails … your actual core job is being in the classroom with students’ (P19, secondary MCT). They missed the pace of the classroom environment. P15 (primary SLT, T3) described how: ‘you don't get that feedback that you usually would as a teacher, to … know, OK, actually all that work I've put in has been worth it … you get the feedback minute by minute almost in the classroom’. There was positivity about a full reopening in September but participants understood that this would not involve a full return to normal and, for most, this was dispiriting.

T4 was characterized by a great deal of pleasure at being back with the children. As P14 (primary MCT, T4) said: ‘That still is a high, actually, physically getting to be with them and I dread the thought of ever having to teach via video or in any other way ever again’. However, they also described how the necessary restrictions made it hard for them to do their jobs as they would have liked, and how working in bubbles (groups of pupils who were not allowed to mix with any other group to restrict COVID infection) and experiences of those bubbles ‘bursting’ (pupils unable to attend in‐person classes as a result of COVID infection within the bubble) tarnished the experience. P11 (primary ECT, T4) described a situation in which a child had split his head open in the playground ‘and it was a really, really bad wound to his forehead and I was delivering first aid and I was on the phone to 999 but the head teacher didn't want to come over because of mixing bubbles and I felt really stressed’. Some also found it hard teaching pupils who had missed so much learning. P8 (secondary SLT, T4) said: ‘we've had to spend a lot of time teaching basic learning, you know, sitting in a classroom and actually learning again, because a lot of them had completely lost it’.

Being back in school with the pupils was some recompense for these challenges but professional pride took a further hit when the government did not include schools and teachers in the second national lockdown, planned for just after the T4 interviews. There were signs that this had a serious negative impact on their professional pride as it suggested the government, and wider society, considered them to be expendable in some way. As P3 (primary SLT, T4) put it: ‘it feels almost like the rest of the country has been asked to stop what they're doing and keep themselves safe but we're still alright to go in and mix with 450 kids, their parents, 70 odd members of staff. It just beggars belief’.

The consistent observation at all time points was that participants were happiest when they were with pupils, interacting and supporting their learning. This was hit from the outset although, at T1, people still thought it would be more temporary than it turned out to be. Ongoing worry about pupils, along with feeling criticized by society, dented professional pride and by T3 many participants were feeling flat. They looked forward to full reopenings in September – and indeed they loved being back in person with pupils – but the restrictions made it difficult to do their jobs in the way they wanted and in the way they felt their pupils deserved. This sense of frustration was made worse by not being included in, or consulted about, a second national lockdown throughout which they would be required to work in schools. It was clear by T4 that professional pleasure and pride in their work had been severely depleted. The exhaustion described in Theme 3, combined with this reduced pleasure and pride left many feeling very ground down.

## DISCUSSION

The pandemic and associated restrictions proved challenging for many individuals, including teachers as they navigated partial school closures, partial school reopenings and full school reopenings during the first 8 months. Indeed, the adverse consequences that UNESCO (2020) predicted – around confusion and stress, challenges in distance learning, and challenges in assessment – were all apparent in the teachers' self‐stories. The four longitudinal themes we have presented show how teachers grew in their frustrations about the uncertainties they experienced and in their concern for pupils, all while becoming increasingly exhausted and less satisfied by the job of teaching in these difficult circumstances. Indeed, these changing and intensified individual, professional and contextual factors appear to have clearly affected teachers' professional identities (Suarez & McGrath, 2022). In turn, this appears to have affected their behaviours and attitudes and their reflections on the educational system, its future and their place within it.

### Teachers' self‐stories

The high‐, low‐ and turning‐point scenes that the participants chose to share with us showed that there were multiple factors contributing to their experiences of being a teacher throughout this time. Contextual factors, including the professional and political environments, can affect teachers' professional identity (Beijaard et al., 2004; Suarez & McGrath, 2022) and it was clear in the stories participants told us that such factors clearly affected how they viewed their profession and their responsibilities. It was clear in our data that the participants saw themselves as planners with strong personal and professional needs to be in control and to work in a structured environment with a clear routine. These needs relate to two of the three basic psychological needs for well‐being and positive functioning identified in Self‐Determination Theory (Deci & Ryan, 1985, 2000, 2002): autonomy, competence and relatedness. The uncertainties they experienced appear to have frustrated participants' autonomy and competence and this was often expressed through anger – even resentment – and navigated by seeking to control what was controllable, for instance by navigating practical concerns around school operations.

Caring for others was an important element of teacher professional identity, and our findings are consistent with other studies (e.g., see Mellon [2022] for a review), attesting to the time‐tested value of this element of teacher identity. Our data showed these teachers to be people who care deeply about their pupils' learning but also their well‐being and safety, which is consistent with previous studies in both pandemic (Jones & Kessler, 2020; Kim et al., 2022) and non‐pandemic times (Klassen et al., 2018). That is, not only do they care and feel responsible for their pupils, but they genuinely like, and draw energy from, the company of children and young people (O'Connor, 2008). Their urge to spend time with pupils is only rivalled by the pleasure they take in interacting with trusted colleagues. Indeed, previous research has found that having positive relationships with colleagues is associated with job satisfaction and decreased intention to leave the profession (Skaalvik & Skaalvik, 2011). Therefore, their inability to interact with and support pupils and their families, and their colleagues fully, especially in times of partial school closures, had a negative impact on their professional identity.

In contrast to the time‐attested element of the caring aspect of teacher professional identity, a new context that many teachers experienced for the first time was remote education. That is, consistent with previous studies during COVID‐19 (e.g., Nazari & Seyri, 2021), teaching remotely was a cause for negotiating what it meant to be a teacher when their pupils were not in the classroom. In England, remote education during the pandemic varied in form, including paper‐based activity packs and online teaching, which elicited varied levels of pupil engagement (Lucas et al., 2020). It was clear that remote education generally did not suit the participants, making them feel less like teachers and more like administrators, and this was not helped by how the wider society was perceived to undervalue the teaching profession at this time (Kim, Oxley, et al., 2021). Moreover, given teachers' need to connect with others in their work, it seems unlikely that remote education would have been sufficiently fulfilling, something that has been reported to be the case for mainstream teachers (Kim et al., 2020; Kim, Leary, et al., 2021; Mellon, 2022) and special education teachers (Schuck & Lambert, 2020). It is possible that if the switch to remote learning was made more permanent, teaching would attract a rather different group of individuals to those it currently attracts. The teachers in our study said they felt like teachers when they were in the classroom or school, interacting intensively with pupils and colleagues, to do a job that goes beyond pedagogy in terms of caring for and supporting pupils to learn.

Moreover, the longitudinal nature of this study provided richness in our understanding of how different factors combine to affect teachers' professional identity. For example, at T3 we saw that teachers became less negative about the government when they perceived the Department for Education as being efficient and respectful of the profession, and of giving them enough time to plan. When this changed again at T4 their resentment and frustration grew and their professional identity was dented. It was notable that teachers were least worried about pupils at T4 when they were back in school, which may indicate that developing systems that allow face‐to‐face (albeit virtual) contact in an equivalent future situation should be a priority for teachers as well as for pupils. We should also consider further research into what works for ensuring both pupil and teacher engagement in remote education. The growing exhaustion of teachers over time may provide us with an indication of where the limit to the job demands teachers can take a lie. We observed teachers coping with a huge amount at T1, and often taking pride in doing so. However, the constant and changing rules and initiatives after the initial crisis seemed to tip the balance on teachers' well‐being, and on professional pride and enjoyment, by T3 and certainly by T4. By observing (dis)continuities over time we can begin to understand the effects that individual, professional and contextual factors can have on teachers' professional identity.

### Implications for supporting teachers

The three teacher‐related adverse consequences of school closures that UNESCO (2020) identified all came to pass between April and November 2020, and it is possible that these may not have been completely avoidable. With the current cost of living crisis in many countries around the world, teachers are again working beyond their ‘traditional’ duties, albeit to a somewhat lesser extent than during national lockdowns. For example, a survey of secondary teachers in the UK found that 88% of teachers recently reported that they or a colleague had provided pupils with toothbrushes and toothpaste, as pupils would not have regular access to such basic resources otherwise (Beauty Banks & British Dental Association, 2023). Given these circumstances, ensuring that sufficient job resources are available to support teachers when facing increased job demands, together with a greater recognition of the value of the teaching profession may help protect and promote teachers' effectiveness and well‐being. As such, we suggest implications for how teachers can be supported in three areas, which are relevant as we emerge from this pandemic.

Teacher confusion and stress were clear from T1 and are illustrated by the theme of uncertainty. Engaging in regular collaborative communications between policymakers and practitioners to communicate significant decisions regarding schools would help reduce uncertainties and organizational planning for teachers.

Moreover, understanding the driving importance of caring for others in teachers' professional identity needs to be recognized and supported. Neglecting to do so may cause teachers to experience fatigue because caring can be costly (Scheffer et al., 2022), especially when not reciprocated by others, including pupils (Noddings, 1992). Therefore, providing reliable and accessible systems and structures where teachers can interact with pupils and colleagues in meaningful ways, may support teachers' positive shaping and perceptions of their professional identity.

The media's portrayal of the teaching profession can be influential in shaping people's views of the profession, and messaging around teachers has become increasingly negative (Ewing et al., 2021). Teachers' work during the pandemic has been particularly scrutinized by the media, and this has negatively affected some teachers (Kim, Oxley, et al., 2021). Therefore, the media should take a less divisive approach to their coverage of the teaching profession as this serves to undermine the goodwill and appreciation in communities, contributing to stress and dissatisfaction for teachers, pupils and families.

### Limitations and future directions

Although this study involved 95 in‐depth interviews, the participants ranged widely in their years of teaching experience and consisted of both classroom teachers and members of SLT, which increased information power (Malterud et al., 2016). Nevertheless, the sample was still small, and findings cannot be generalized to other teachers in England or beyond, or to other contexts such as Alternative Provision settings, though this was not the aim of the current study. Rather, we aimed to shed light on what being a teacher during COVID‐19 was like for the participants, which provides ecological validity and arguably flexible generalisability (Braun & Clarke, 2021).

It could be useful to use these findings as the basis for a quantitative follow‐up study, with a representative sample of teachers in England and other countries. However, it is important to note that the qualitative approach used in the current study provided access to a richness of experience and professional reflection that would not be possible with a quantitative design. As such, there would be value in continuing to work to understand what the COVID‐19 experience can tell us about teachers and teaching using both quantitative and qualitative approaches.

## AUTHOR CONTRIBUTIONS

**Lisa E. Kim:** Conceptualization; funding acquisition; investigation; methodology; project administration; supervision; writing – original draft; writing – review and editing. **Diana Fields:** Formal analysis; writing – original draft; writing – review and editing. **Kathryn Asbury:** Conceptualization; funding acquisition; investigation; methodology; supervision; writing – original draft; writing – review and editing.

## CONFLICT OF INTEREST STATEMENT

None to declare.

## Data Availability

The anonymised data that supports the findings of the current study can be made available to researchers from the corresponding author upon reasonable request.
